# Impact of SARS‐CoV‐2 Pandemic on Emergency Hospitalizations for Acute Respiratory Infections: The Experience of a Paediatric Tertiary Care Hospital in Italy

**DOI:** 10.1111/irv.13335

**Published:** 2024-06-18

**Authors:** Marta Luisa Ciofi degli Atti, Flavia Beccia, Carmen D'Amore, Lucilla Ravà, Paola Bernaschi, Cristina Russo, Alberto Villani, Carlo Federico Perno, Massimiliano Raponi

**Affiliations:** ^1^ Epidemiology, Clinical Pathways and Clinical Risk Unit Bambino Gesù Children's Hospital, IRCCS Rome Italy; ^2^ University Department of Life Sciences and Public Health Università Cattolica del Sacro Cuore Rome Italy; ^3^ Laboratory Medicine Clinical Area Bambino Gesù Children's Hospital, IRCCS Rome Italy; ^4^ University‐Hospital Paediatric Clinical Area Bambino Gesù Children's Hospital, IRCCS Rome Italy; ^5^ Medical Direction Bambino Gesù Children's Hospital, IRCCS Rome Italy

**Keywords:** acute respiratory infections, children, COVID‐19 pandemic, hospitalizations, RSV, viral infections

## Abstract

**Background:**

Acute respiratory infections (ARIs) are a major healthcare issue in children. The SARS‐CoV‐2 pandemic changed the epidemiology of ARIs; the aims of this study are to characterize the epidemiological trend of ARI emergency hospitalizations and virology results and to estimate the association of ARI emergency hospitalizations with respiratory viruses from January 2018 to June 2023.

**Methods:**

This study was carried out in an Italian tertiary care children's hospital (Bambino Gesù Children's Hospital). The demographic and clinical information of children who accessed the Emergency Department (ED) with ARI and were hospitalized were retrospectively extracted from the electronic health records. Multivariate linear regression model was used to compare the number of ARI hospital admissions with the reported temporal trends in viruses diagnosed from respiratory samples throughout the same time period.

**Results:**

During the study period, there were 92,140 ED visits and 10,541 hospitalizations due to ARIs, reflecting an admission rate of 11.4%. The highest proportion of hospitalizations occurred in infants ≤ 1 year of age (*n* = 4840, 45.9% of total admissions), with a hospitalization rate of 22.6%. Emergency hospitalizations aligned closely with the predictions made by the multivariate regression model; peaks in hospitalizations reflected Respiratory Syncytial Virus (RSV) circulation.

**Conclusions:**

ARI hospital urgent admissions are a relevant component of ARI disease burden in children. RSV prevention and control are crucial to limit the risk of urgent hospitalizations due to ARIs.

## 1 Introduction

Acute respiratory infections (ARIs) are one of the main causes of mortality and morbidity in children [1, 2, 3] causing more than 12 million annual hospital admissions and affecting mostly children aged less than 5 years [4, 5]. The majority of ARI in children are caused by viruses, such as rhinovirus, respiratory syncytial virus (RSV), influenza and parainfluenza viruses, enteroviruses, adenoviruses and metapneumoviruses [6, 7, 8, 9]. Prior to the SARS‐CoV‐2 pandemic, these infections were characterized by a marked seasonality, with peaks of incidence in the autumn–winter period.

Italy was the first European country hit by the COVID‐19 pandemic, with the first autochthonous patient diagnosed on 20 February 2020 [10]. Public health measures introduced to contain the pandemic [11, 12] limited the transmission of respiratory viruses [13, 14], interfering with their seasonality and reducing emergency department (ED) visits and hospitalizations [15, 16]. With the reduction of social isolation measures in 2021–2022, there was a resurgence of ARI cases and a different seasonality of RSV, with an earlier peak and shorter duration than in pre‐pandemic seasons [17, 18]. Regression models showed that peaks of ED ARI visits corresponded to the peaks of influenza, RSV and rhinovirus in the prepandemic seasons (2018–2019 and 2019–2020), to the peaks of SARS‐CoV‐2 and rhinovirus in 2020 and to the peaks of RSV, parainfluenza and rhinovirus in 2021 and 2022 [19].

Clinical severity of ARIs varies by pathogen [20], with different risks of hospitalization. The aims of this study were to characterize the epidemiological trend of ARI emergency hospital admissions and virology results from January 2018 to June 2023 and to estimate the association of ARI emergency hospitalizations with respiratory viruses.

## 2 Methods

### 2.1 Setting

This study was carried out at Bambino Gesù Children's Hospital (OPBG), a tertiary care academic hospital with 607 inpatient beds, which provides its care activities in Rome, Italy. The ED provides free urgent medical care to the paediatric population on a 24/7 basis.

Total accesses to ED were 85,012 in 2018 and 89,558 in 2019; in 2020, they decreased to 62,010, to increase to 79,624 in 2021 and to 95,351 in 2022. In the first semester of 2023, there were 53,662 ED visits. In the study period, about 20% of ED visits was due to ARIs [19].

### 2.2 Data Collection

Demographic and clinical information of children who accessed the ED and were diagnosed with ARI from 1 January 2018 to 30 June 2023 was retrospectively extracted from computerized ED reports. In detail, information on the patient's demographics, ICD‐9‐CM diagnosis and status at discharge (i.e., hospitalized or discharged home) was anonymized and inferred. ARI was defined based on the ICD‐9 CM diagnosis at discharge from the ED (Supporting Information S1).

Information on the virologic results of respiratory specimens (e.g., nasopharyngeal swabs, tracheal swabs and/or bronchoalveolar lavages) obtained in the same time period from OPBG patients was extracted from the from the Hospital Electronic Laboratory Information System. Respiratory samples that tested positive for the same virus within 3 months were excluded from the analysis.

### 2.3 Laboratory Testing

Respiratory specimens were tested with multiple‐target RT‐PCR assays capable of simultaneously detecting 18 respiratory viruses, including RSV A and B, influenza A and B viruses, human coronavirus OC43, 229E, NL‐63 and HUK1, adenovirus, human rhinovirus (hRV), parainfluenza virus 1‐2‐3‐4, human metapneumovirus‐hMPV, human bocavirus‐hBoV, and enterovirus, through the commercial multiplex RT‐PCR (AllplexTM Respiratory Panel Assay Seegene Korea) kit. Nucleic acids were extracted using a combined one‐step extraction and PCR setting from a 200‐μL sample of respiratory samples loaded directly onto an automated instrument using ready‐to‐use extraction reagent cassettes (StarMag universal cartridges on Seegene Starlet) and amplified on the CFX96TM real‐time PCR detection system (BioRad Laboratories). Antigen test results were also considered for the diagnosis of RSV infection. As of 2020, SARS‐CoV‐2 molecular tests or SARS‐CoV‐2 antigen tests have also been performed.

### 2.4 Statistical Analysis

The numbers of ED visits, emergency hospitalizations and positive virological tests by year, week and age group (< 1 year, 1–4 years, 5–9 years, ≥ 10 years) were summarized as counts and percentages; positive tests were also described by type of virus. The significance of time trends was assessed using the Cochrane Armitage test.

The time trends observed in viruses diagnosed from respiratory samples were compared with the number of emergency hospitalizations in the time frame January 2018–June 2023.

The number of hospitalizations was considered the dependent variable in a multiple linear regression models, whose independent variables were the weekly number of positive laboratory results for a single virus.

The expected number of hospitalizations in a weekly period was estimated with the following formula:

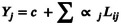

where *L*_*ij*_ is the number of positive samples for virus *i* in week *j*, ∝_*i*_ is the regression coefficient for virus *i* used to estimate the number of hospital admissions associated with each virus and *c* is the constant representing the number of hospitalizations that could be attributed to viruses not included in the model.

All viruses that had a *p*‐value < 0.2 in the univariate models were included in the multivariate models. On the other hand, viruses that had a negative regression coefficient in univariate models (which is biologically implausible) were excluded. The validity of the final model was assessed in terms of the proportion of the variation it explained (*R*‐squared), the significance of the joint relationship between the observed and independent variables in each model (i.e., the probability that R differed significantly from zero) and the impact of changes in the model specification and the relative amount of information lost by a given model (AIC, Akaike information criteria). We also computed specific multivariate linear regression models for age classes (≤ 1 year, 1–4 years, ≥ 5 years). The attribution of the ARI rate to each virus was generated by multiplying each regression coefficient by its corresponding virus count. The rate of unattributed ARI to viruses included in the models may be caused by other viruses or bacterial infections. These cases are included in the term ‘unattributed’ in model descriptions and representations. The weekly number of respiratory viruses used in the models are reported in Supporting Information S2.

All statistical analyses were performed using STATA 17 Software (StataCorp LP, College Station, TX).

## 3 Results

During the study period, there were 92,140 ED visits and 10,541 hospitalizations related to ARI, with an admission rate of 11.4%. The highest proportion of hospitalizations occurred in infants ≤ 1 year (*n* = 4840, 45.9% of total admissions), with a hospitalization rate of 22.6% (Table 1). The hospitalization rate was 6.5% in children aged 1–4 years, 7.9% in children aged 5–9 years and 14.7% in children aged ≥10 years. Proportion of hospitalizations significantly changed over the study period, with a decline in 2021 and 2022 (10.6% and 8.5%) (Table 1).

**TABLE 1:** ED visits and hospital admissions by ARI by age group and year; OPBG, January 2018–June 2023.

Age group	2018		2019		2020		2021		2022		2023		2018–2023		Trend *p*‐value	Trend *p*‐value
	ED visits *N* (%)	Admissions *N* (% of visits)	ED visits *N* (%)	Admissions *N* (% of visits)	ED visits *N* (%)	Admissions *N* (% of visits)	ED visits *N* (%)	Admissions *N* (% of visits)	ED visits *N* (%)	Admissions *N* (% of visits)	ED visits *N* (%)	Admissions *N* (% of visits)	ED visits *N* (%)	Admissions *N* (% of visits)	ED visits	Admissions
< 1 year	4517 (26.5)	1201 (26.6)	4586 (24.5)	1175 (25.6)	2666 (20.6)	660 (24.8)	3611 (22.4)	745 (20.6)	4170 (22.9)	665 (15.9)	1858 (20.2)	394 (21.2)	21,408 (23.0)	4840 (22.6)	< 0.001	< 0.001
1–4 years	8085 (47.5)	579 (7.2)	8743 (46.7)	589 (6.7)	5476 (42.4)	435 (7.9)	8923 (55.4)	497 (5.6)	8864 (48.7)	457 (5.2)	4061 (44.2)	314 (7.7)	44,152 (48.3)	2871 (6.5)	< 0.001	< 0.001
5–9 years	2748 (16.1)	258 (9.4)	3324 (17.8)	266 (8.0)	2662 (20.6)	213 (8.0)	1873 (11.6)	151 (8.1)	2925 (16.1)	183 (6.3)	2182 (23.8)	163 (7.5)	15,714 (16.3)	1234 (7.9)	< 0.001	< 0.001
≥ 10 years	1679 (9.9)	270 (16.1)	2064 (11.0)	302 (14.6)	2119 (16.4)	319 (15.1)	1692 (10.5)	321 (19.0)	2227 (12.2)	242 (10.9)	1085 (11.8)	142 (13.1)	10,866 (11.8)	1596 (14.7)	< 0.001	< 0.001
Total	17,029 (100.0)	2308 (13.6)	18,717 (100.0)	2332 (12.5)	12,923 (100.0)	1627 (12.6)	16,099 (100.0)	1714 (10.6)	18,186 (100.0)	1547 (8.5)	9186 (100.0)	1013 (11.0)	92,140 (100.0)	10,541 (11.4)	< 0.001	< 0.001

*Note:* For 2023, January–June data are provided.

**FIGURE 1:**
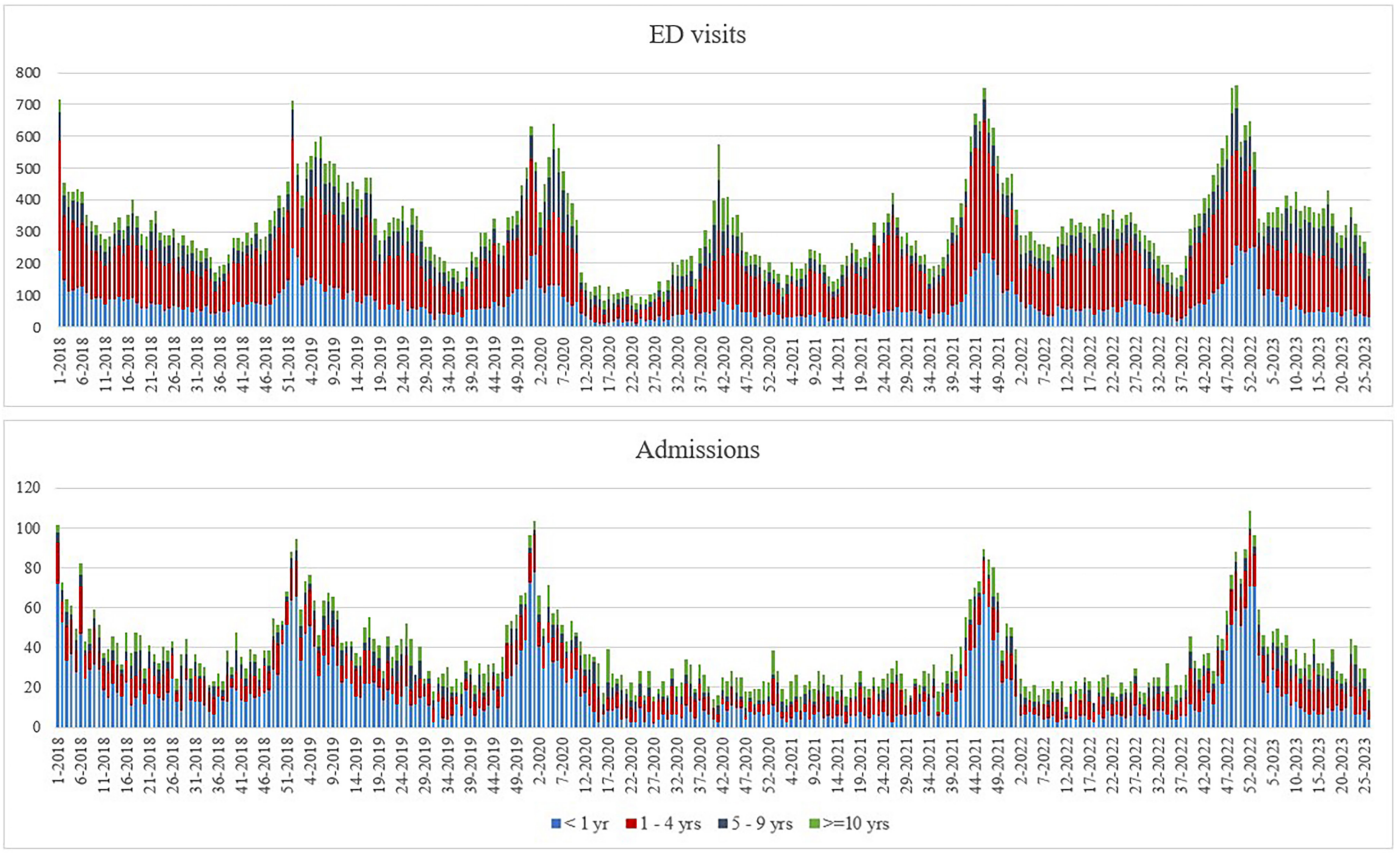
Number of ED visits and hospital admissions for ARI, by week and age group, registered at OPBG, January 2018–June 2023.

The weekly trend analysis shows that in years 2018–2020, the peak of ED visits occurred in January, with about 700 weekly visits in 2018 and 2019 and over 600 in 2020. The number of ED visits sharply decreased from the beginning of national lockdown in March 2020, remaining lower than prepandemic levels until October 2021, when a significant increase was observed. In December 2021 and 2022, ED visit peaks were higher than in the prepandemic period (over 750 weekly visits) (Figure 1).

The impact of the pandemic on ARI hospitalizations was more pronounced. In years 2018–2020, there was a maximum of about 100 weekly admissions in January, coinciding with the peak of ED visits. Hospitalizations were fewer than 40 per week from March 2020 to September 2021. In subsequent seasons, seasonal peaks were observed, reaching the maximum of 108 weekly hospitalizations in January 2023 (Figure 1).

In the prepandemic period, the majority of viral infections confirmed from respiratory samples were rhinoviruses (> 35%), followed by RSV (> 18%) and influenza, parainfluenza and adenovirus viruses (≥ 7%). In years 2020 to 2022, the most frequent virus was SARS‐CoV‐2, showing an increasing trend over the 3 years (44%, 46% and 60%). In 2023, rhinoviruses returned to being most frequent (24.8%), followed by adenovirus (13%) and SARS‐CoV‐2 (11.4%) (Table 2).

**TABLE 2:** Distribution of viral respiratory infections by year registered at OPBG, January 2018–June 2023.

Virus	2018		2019		2020		2021		2022		2023		2018–2023		Trend *p*‐value
	*N*	%	*N*	%	*N*	%	*N*	%	*N*	%	*N*	%	*N*	%	
SARS‐CoV‐2	—	—	—	—	2159	43.8	3737	46.4	7217	60.0	462	11.4	13,575	34.6	< 0.001
Rhinovirus	1828	35.9	1809	35.5	998	20.2	956	11.9	1418	11.8	1001	24.8	8010	20.4	< 0.001
RSV	990	19.4	937	18.4	382	7.7	988	12.3	673	5.6	265	6.6	4235	10.8	< 0.001
Influenza	401	7.9	588	11.5	588	11.9	9	0.1	439	3.6	299	7.4	2324	5.9	< 0.001
Parainfluenza	440	8.6	354	6.9	86	1.7	596	7.4	563	4.7	299	7.4	2338	6.0	< 0.001
Adenovirus	345	6.8	399	7.8	196	4.0	279	3.5	391	3.2	512	12.7	2122	5.4	0.410
Bocavirus	313	6.1	276	5.4	164	3.3	291	3.6	281	2.3	252	6.2	1577	4.0	< 0.001
Coronavirus	281	5.5	262	5.1	173	3.5	311	3.9	272	2.3	282	7.0	1581	4.0	< 0.001
Human rhinovirus/enterovirus	0	0.0	0	0.0	15	0.3	699	8.7	366	3.0	209	5.2	1289	3.3	< 0.001
Enterovirus	321	6.3	277	5.4	110	2.2	27	0.3	249	2.1	183	4.5	1167	3.0	< 0.001
Human metapneumovirus	174	3.4	197	3.9	62	1.3	162	2.0	169	1.4	278	6.9	1042	2.7	0.688
Total	5093	100.0	5099	100.0	4933	100.0	8055	100.0	12,038	100.0	4042	100.0	39,260	100.0	

*Note:* For 2023, January–June data are provided.

**FIGURE 2:**
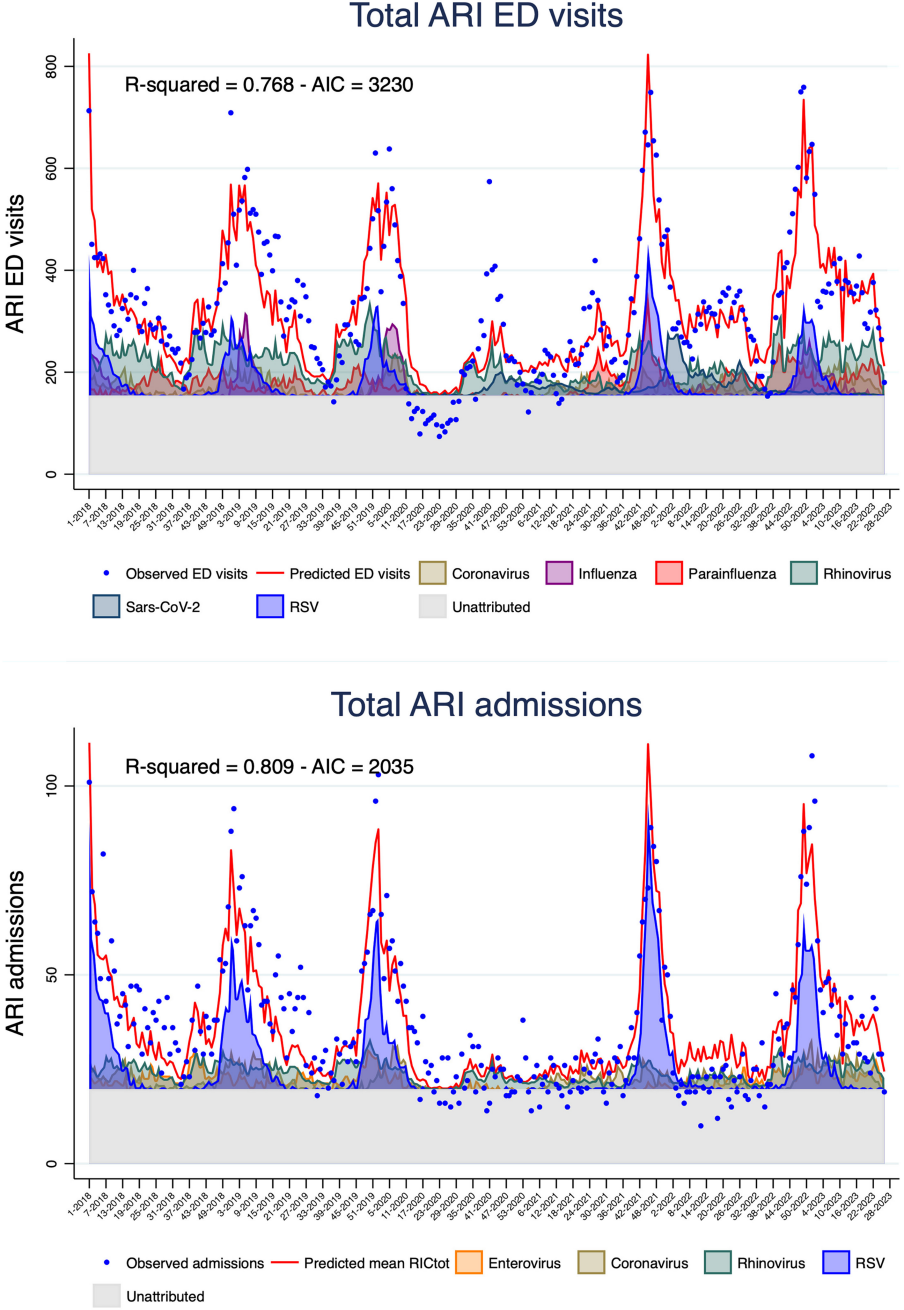
Multivariate linear regression model for weekly emergency hospital admissions for ARI; OPBG, January 2018–June 2023.

**FIGURE 3:**
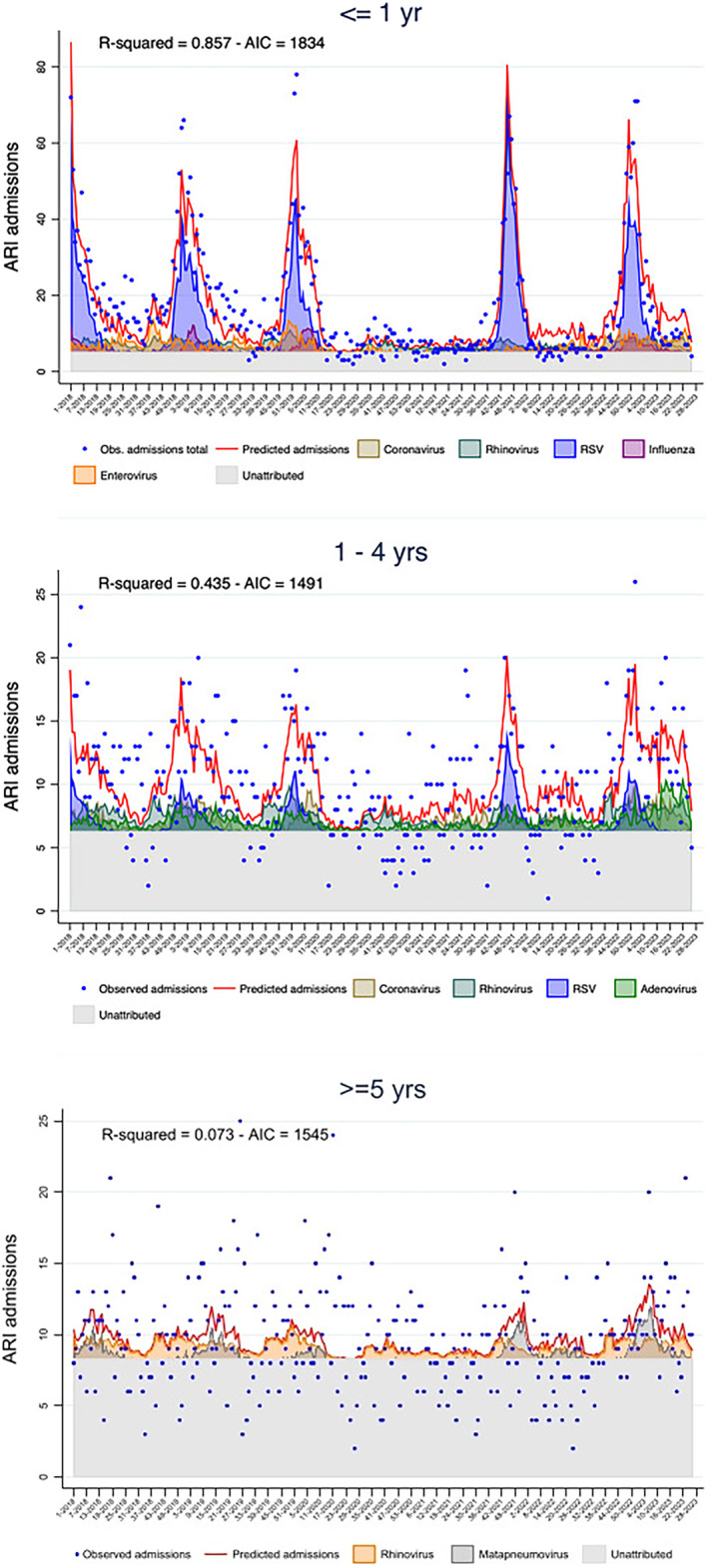
Multivariate linear regression models for weekly emergency hospital admissions for ARI, by age groups; OPBG, January 2018–June 2023.

The seasonal pattern of the five most frequently diagnosed viruses from respiratory samples (SARS‐CoV‐2, rhinovirus, RSV, influenza and parainfluenza) is illustrated in Supporting Information S2: Figures 1–5.

SARS‐CoV‐2 was initially diagnosed in March 2020 and peaked between December 2021 and March 2022, with a second peak in June of the same year (Supporting Information S2: Figure 1). Rhinoviruses showed a recovery of seasonality observed in the pre‐pandemic period, after a significant decrease observed between March and July 2020 (Supporting Information S2: Figure 2). RSV and influenza seasonal pattern were interrupted in 2020. In December 2021, RSV reached its highest observed peak (Supporting Information S2: Figure 3); throughout the study period, RSV mostly affected children up to 5 years of age. Influenza resumed a seasonal pattern in 2022–2023 (Supporting Information S2: Figure 4). Parainfluenza viruses had a higher incidence in the post‐pandemic period than in the prepandemic period (Supporting Information S2: Figure 5). Fewer cases of adenovirus (Supporting Information S2: Figure 6), bocavirus (Supporting Information S2: Figure 7), coronavirus (Supporting Information S2: Figure 8), enterovirus (Supporting Information S2: Figure 9) and metapneumovirus (Supporting Information S2: Figure 10) were registered, reflecting a return to seasonal patterns in 2022–2023.

Emergency hospitalizations aligned closely with the predictions made by the multivariate regression model (*R*‐squared value: 0.809) (Figure 2). The model overestimated cases from February to June 2022.

Viruses included in the model were enteroviruses, coronaviruses other than SARS‐CoV‐2, rhinoviruses and RSV. In the model, the peaks in hospitalizations reflected RSV circulation. Considering all age groups, the model attributed 18% of ARI hospitalizations to RSV, 13% to rhinovirus, 8% to coronaviruses other than SARS‐CoV‐2 and 7% to enterovirus (Supporting Information S2: Table 1).

Within specific age groups, the model had a *R*‐squared value of 0.857 in infants aged ≤ 1 year. As in the model including the whole population, ARI hospitalizations in infants peak mirrored RSV circulation patterns (Figure 3). The model attributed 36% of cases to RSV, 12% to rhinovirus, 11% to enterovirus, 6% to coronaviruses other than SARS‐CoV‐2 and 4% to influenza (Supporting Information S2: Table 1).

In other age groups, the model goodness of fit was lower, with *R*‐squared values < 0.5 (*R*‐squared 0.435 in the 1–4 age group and 0.073 in the ≥ 5 age group). RSV, adenovirus, coronaviruses other than SARS‐CoV‐2 and rhinovirus were included in the 1–4 years group model and rhinovirus and metapneumovirus in the older age group (Figure 3).

## 4 Discussion

This study shows that emergency hospitalizations are a relevant component of ARI burden in children; most of the about 10 thousand hospitalizations regarded infants (45.9%), as expected given the highest disease severity in this age group [20, 21]. There are few data in literature describing the rate of ARI hospitalizations in children calculated over ARI ED accesses [22]. In our experience, approximately 10% of children presenting with ARI at the ED required hospitalization. The substantial proportion of children discharged home suggests possible ED inappropriate referral, since ARIs with mild clinical characteristics should be treated in outpatient settings [23].

The use of ED services does vary by country, as the structure of healthcare differs in terms of payment and organization [24, 25]. In Italy, emergency visits and hospitalizations are free of charge, and primary care paediatricians and general practitioners provide free of charge care in the outpatient setting. ED use in children may differ across the country, given the regional organization of health services and the differences in access to care across regions [26, 27].

In our setting, improving integration with primary care paediatricians emerges as pivotal for mitigating the ED overuse for ARI. Family‐focused health education and health literacy initiatives can also improve parental knowledge of indications for ED referral, since low parental health literacy is an independent predictor of increased ED recourse, especially for children without chronic diseases [28, 29, 30, 31].

Weekly trend analysis confirmed that ARI hospitalizations were deeply influenced by the SARS‐CoV‐2 pandemic and its containment measures.

RSV was the predominant cause of emergency hospitalizations due to ARIs, had a marked pre‐pandemic seasonality [8] that was interrupted since March 2020 to September 2021, followed by a peak in late winter 2021 and a return to prepandemic seasonal incidence in 2022 [32].

Other viruses related to hospitalizations were enteroviruses, rhinovirus and coronavirus other than SARS‐CoV‐2, all of which were disrupted in various ways by the pandemic [33, 34, 35, 36, 37, 38].

Influenza was related to hospitalizations in infants and, as shown by other authors, had an abrupt and premature conclusion in the 2019–2020 season [39] and a persistently low activity into the 2021–2022 season [40, 41, 42, 43, 44], possibly due to mask‐wearing, social distancing and reduction in international travels [14]. The seasonal peak we observed in 2022–2023 season was in line with data from national virological surveillance [45].

SARS‐CoV‐2 circulation did not reflect on hospitalizations, which is not surprising since SARS‐CoV‐2 in children is rarely severe [46].

In this study, the regression model estimates of ARI hospitalizations corresponded closely with the observed weekly numbers; we documented that almost 70% of the ARI hospitalizations can be attributed to a viral infection. The proportion of ARI hospitalizations that was not attributed to viruses included in the model may be caused by other viruses or to bacterial infections.

Hospitalizations by age estimated by regression models were in line with observed cases in infants ≤ 1 year of age that represented approximately 50% of all ARI hospitalizations over the study period. In older children, the models did not explain the observed hospitalisations; this finding could be due to the limited numbers of observations and to the role of variables other than viral infections, such as comorbidities.

The findings of this study should be interpreted within its limitations and strengths. The study was conducted in a tertiary care academic hospital, potentially limiting its generalizability to other contexts. In addition, clinical presentation and coinfections could had a role in explain hospitalizations, particularly in children older than 1 year where the goodness‐of‐fit of the regression models was lower. Despite possible limitations, our findings show robust data over a period of more than 5 years; all patients received a diagnosis of ARI by paediatricians of a tertiary care academic children's hospital, and we provided an extended analysis of viruses causing ARI in children.

As shown in this study, RSV prevention is crucial to limit ARI hospitalizations. Pharmacological prevention of RSV involves the administration of monoclonal antibodies (such as palivizumab and nirsevimab) in high‐risk children up to 2 years of age [47, 48]. Recent authorization of RSV vaccine to protect infants up to 6 months of age prospects for a future vaccination strategy in paediatric populations [49, 50].

## Author Contributions

**Marta Luisa Ciofi degli Atti:** supervision, writing–original draft, writing–review and editing, conceptualization, funding acquisition. **Flavia Beccia:** writing–original draft, data curation, writing–review and editing. **Carmen D'Amore:** data curation, writing–review and editing. **Lucilla Ravà:** formal analysis, data curation, writing–review and editing, methodology. **Paola Bernaschi:** writing–review and editing. **Cristina Russo:** writing–review and editing. **Alberto Villani:** writing–review and editing, supervision. **Carlo Federico Perno:** writing–review and editing, funding acquisition, supervision. **Massimiliano Raponi:** supervision, writing–review and editing, project administration, funding acquisition.

## Ethics Statement

The study was approved by the Ethics Committee of the Bambino Gesù Children's Hospital (3072_OPBG_2023).

## Consent

Considering the retrospective study design, written informed consent was not deemed necessary.

## Conflicts of Interest

The authors declare no conflicts of interest.

### Peer Review

The peer review history for this article is available at https://www.webofscience.com/api/gateway/wos/peer‐review/10.1111/irv.13335.

## Supporting information


**Data S1.** ICD9‐CM encodings for the diagnosis of ARI.

**Figure S1.** Number of SARS‐CoV‐2 positive respiratory specimens per week and age group registered at OPBG, January 2018–June 2023.
**Figure S2.** Number of rhinovirus‐positive respiratory specimens per week and age group registered at OPBG, January 2018–June 2023.
**Figure S3.** Number of RSV positive respiratory specimens per week and age group registered at OPBG, January 2018–June 2023.
**Figure S4.** Number of positive respiratory samples for influenza viruses by week and age group registered at OPBG, January 2018–June 2023.
**Figure S5.** Number of positive respiratory samples for parainfluenza viruses per week and age group registered at OPBG, January 2018–June 2023.
**Figure S6.** Number of positive respiratory samples for Adenovirus per week and age group registered at OPBG, January 2018–June 2023.
**Figure S7.** Number of positive respiratory samples for Bocavirus per week and age group registered at OPBG, January 2018–June 2023.
**Figure S8.** Number of positive respiratory samples for Coronavirus per week and age group registered at OPBG, January 2018–June 2023.
**Figure S9.** Number of positive respiratory samples for Enterovirus per week and age group registered at OPBG, January 2018–June 2023.
**Figure S10.** Number of positive respiratory samples for Metapneumovirus per week and age group registered at OPBG, January 2018–June 2023.
**Table S1.** Percentages of ARI emergency hospital admissions attributed to different respiratory virus counts by age classes.

## Data Availability

The data that support the findings of this study are available from the corresponding author upon reasonable request.
