# Supplementary material for: Impact of SARS‐CoV‐2 Pandemic on Emergency Hospitalizations for Acute Respiratory Infections: The Experience of a Paediatric Tertiary Care Hospital in Italy
Source: Influenza Other Respir Viruses. 2024 Jun 18;18(6):e13335. doi: 10.1111/irv.13335 (PMC11184210; doi:10.1111/irv.13335)
Supplement: Supplementary file 1 — Data S1. ICD9‐CM encodings for the diagnosis of ARI. [file IRV-18-e13335-s002.docx]

SUPPLEMENTARY FILE 1. ICD9- CM Encodings for the Diagnosis of ARI

| **ICD-9 CM code** | **Description** |
| --- | --- |
| 460 | ACUTE NASOPHARYNGITIS |
| 461 | AC SINUSITIS |
| 4610 | AC MAXILLARY SINUSITIS |
| 4611 | AC FRONTAL SINUSITIS |
| 4612 | AC ETHMOIDAL SINUSITIS |
| 4613 | AC SPHENOIDAL SINUSITIS |
| 4618 | OTHER ACUTE SINUSITIS |
| 4619 | ACUTE SINUSITIS NOS |
| 462 | ACUTE PHARYNGITIS |
| 463 | ACUTE TONSILLITIS |
| 4640 | AC LARYNGITIS |
| 46400 | AC LARYNGITIS W/O OBST |
| 46401 | AC LARYNGITIS W OBSTRUCT |
| 46410 | AC TRACHEITIS NO OBSTRUC |
| 46411 | AC TRACHEITIS W OBSTRUCT |
| 4642 | AC LARYNGOTRACH |
| 46420 | AC LARYNGOTRACH NO OBSTR |
| 46421 | AC LARYNGOTRACH W OBSTR |
| 4643 | AC EPIGLOTTITIS |
| 46430 | AC EPIGLOTTITIS NO OBSTR |
| 46431 | AC EPIGLOTTITIS W OBSTR |
| 4644 | CROUP |
| 4659 | ACUTE URI NOS |
| 4660 | ACUTE BRONCHITIS |
| 46611 | ACU BRONCHOLITIS D/T RSV |
| 46619 | ACU BRNCHLTS D/T OTH ORG |
| 4800 | ADENOVIRAL PNEUMONIA |
| 4801 | RESP SYNCYT VIRAL PNEUM |
| 4802 | PARINFLUENZA VIRAL PNEUM |
| 4808 | VIRAL PNEUMONIA NEC |
| 4809 | VIRAL PNEUMONIA NOS |
| 481 | PNEUMOCOCCAL PNEUMONIA |
| 48283 | PNEUMO OTH GRM-NEG BACT |
| 48289 | PNEUMONIA OTH SPCF BACT |
| 4829 | BACTERIAL PNEUMONIA NOS |
| 4830 | PNEU MYCPLSM PNEUMONIAE |
| 4838 | PNEUMON OTH SPEC ORGNSM |
| 485 | BRONCHOPNEUMONIA ORG NOS |
| 486 | PNEUMONIA, ORGANISM NOS |
| 4870 | INFLUENZA WITH PNEUMONIA |
| 4871 | FLU W RESP MANIFEST NEC |
| 4878 | FLU W MANIFESTATION NEC |
| 7806 | FEVER |
| 78606 | TACHYPNEA |
| 78609 | RESPIRATORY ABNORM NEC |
| 7862 | COUGH |
| 78650 | CHEST PAIN NOS |
| 78651 | PRECORDIAL PAIN |
| 78652 | PAINFUL RESPIRATION |
| 78659 | CHEST PAIN NEC |
| 7931 | ABN FINDINGS-LUNG FIELD |
