# Supplementary material for: Impact of SARS‐CoV‐2 Pandemic on Emergency Hospitalizations for Acute Respiratory Infections: The Experience of a Paediatric Tertiary Care Hospital in Italy
Source: Influenza Other Respir Viruses. 2024 Jun 18;18(6):e13335. doi: 10.1111/irv.13335 (PMC11184210; doi:10.1111/irv.13335)
Supplement: Supplementary file 2 — Figure S1. Number of SARS‐CoV‐2 positive respiratory specimens per week and age group registered at OPBG, January 2018–June 2023. Figure S2. Number of rhinovirus‐positive respiratory specimens per week and age group registered at OPBG, January 2018–June 2023. Figure S3. Number of RSV positive respiratory specimens per week and age group registered at OPBG, January 2018–June 2023. Figure S4. Number of positive respiratory samples for influenza viruses by week and age group registered at OPBG, January 2018–June 2023. Figure S5. Number of positive respiratory samples for parainfluenza viruses per week and age group registered at OPBG, January 2018–June 2023. Figure S6. Number of positive respiratory samples for Adenovirus per week and age group registered at OPBG, January 2018–June 2023. Figure S7. Number of positive respiratory samples for Bocavirus per week and age group registered at OPBG, January 2018–June 2023. Figure S8. Number of positive respiratory samples for Coronavirus per week and age group registered at OPBG, January 2018–June 2023. Figure S9. Number of positive respiratory samples for Enterovirus per week and age group registered at OPBG, January 2018–June 2023. Figure S10. Number of positive respiratory samples for Metapneumovirus per week and age group registered at OPBG, January 2018–June 2023. Table S1. Percentages of ARI emergency hospital admissions attributed to different respiratory virus counts by age classes. [file IRV-18-e13335-s001.docx]

SUPPLEMENTARY FILE 2.

Figure 1. Number of SARS-CoV-2 positive respiratory specimens per week and age group registered at OPBG, January 2018-June 2023

Figure 2. Number of rhinovirus-positive respiratory specimens per week and age group registered at OPBG, January 2018-June 2023

Figure 3. Number of RSV positive respiratory specimens per week and age group registered at OPBG, January 2018-June 2023.

Figure 4. Number of positive respiratory samples for influenza viruses by week and age group registered at OPBG, January 2018-June 2023

Figure 5. Number of positive respiratory samples for parainfluenza viruses per week and age group registered at OPBG, January 2018-June 2023

Figure 6. Number of positive respiratory samples for Adenovirus per week and age group registered at OPBG, January 2018-June 2023

Figure 7. Number of positive respiratory samples for Bocavirus per week and age group registered at OPBG, January 2018-June 2023

Figure 8. Number of positive respiratory samples for Coronavirus per week and age group registered at OPBG, January 2018-June 2023

Figure 9. Number of positive respiratory samples for Enterovirus per week and age group registered at OPBG, January 2018-June 2023

Figure 10. Number of positive respiratory samples for Metapneumovirus per week and age group registered at OPBG, January 2018-June 2023

Table 1. Percentages of ARI emergency hospital admissions attributed to different respiratory virus counts by age class

| **Respiratory virus** | **Total population** | **<1 yr** | **1 - 4 yrs** | **≥ 5 yrs** |
| --- | --- | --- | --- | --- |
| **Enterovirus** | 7% | 11% | - | - |
| **Adenovirus** | - | - | 9% | - |
| **RSV** | 18% | 36% | 7% | - |
| **Coronavirus** | 8% | 6% | 8% | - |
| **Metapneumovirus** | - | - | - | 5% |
| **Influenza** | - | 4% | - | - |
| **Rhinovirus** | 13% | 12% | 13% | 9% |
| **Unattributed** | 54% | 31% | 63% | 86% |
